# Grasp Stability Prediction for a Dexterous Robotic Hand Combining Depth Vision and Haptic Bayesian Exploration

**DOI:** 10.3389/frobt.2021.703869

**Published:** 2021-08-12

**Authors:** Muhammad Sami Siddiqui, Claudio Coppola, Gokhan Solak, Lorenzo Jamone

**Affiliations:** ARQ (Advanced Robotics at Queen Mary), School of Electronic Engineering and Computer Science, Queen Mary University of London, London, United Kingdom

**Keywords:** grasping, manipulation, dexterous hand, haptics, grasp metric, exploration

## Abstract

Grasp stability prediction of unknown objects is crucial to enable autonomous robotic manipulation in an unstructured environment. Even if prior information about the object is available, real-time local exploration might be necessary to mitigate object modelling inaccuracies. This paper presents an approach to predict safe grasps of unknown objects using depth vision and a dexterous robot hand equipped with tactile feedback. Our approach does not assume any prior knowledge about the objects. First, an object pose estimation is obtained from RGB-D sensing; then, the object is explored haptically to maximise a given grasp metric. We compare two probabilistic methods (i.e. standard and unscented Bayesian Optimisation) against random exploration (i.e. uniform grid search). Our experimental results demonstrate that these probabilistic methods can provide confident predictions after a limited number of exploratory observations, and that unscented Bayesian Optimisation can find safer grasps, taking into account the uncertainty in robot sensing and grasp execution.

## 1 Introduction

Autonomous robotic grasping of arbitrary objects is a challenging problem that is becoming increasingly popular in the research community due to its importance in several applications, such as pick-and-place in manufacturing and logistics, service robots in healthcare and robotic operations in hazardous environments, e.g. nuclear decommissioning (Billard and Kragic, 2019; Graña et al., 2019). Grasping involves several phases: from detecting the object location to choosing the grasp configuration (i.e. how the gripper or robot hand should contact the object) with the final objective of keeping the object stable in the robot grip. Moreover, when we consider dexterous robotic hands with multiple fingers, several contact points on an object must be identified to achieve a robust grasp (Miao et al., 2015; Ozawa and Tahara, 2017). This is particularly challenging when limited or no prior information is available about the object, and therefore it is necessary to rely more heavily on real-time robot perception.

**FIGURE 1:**
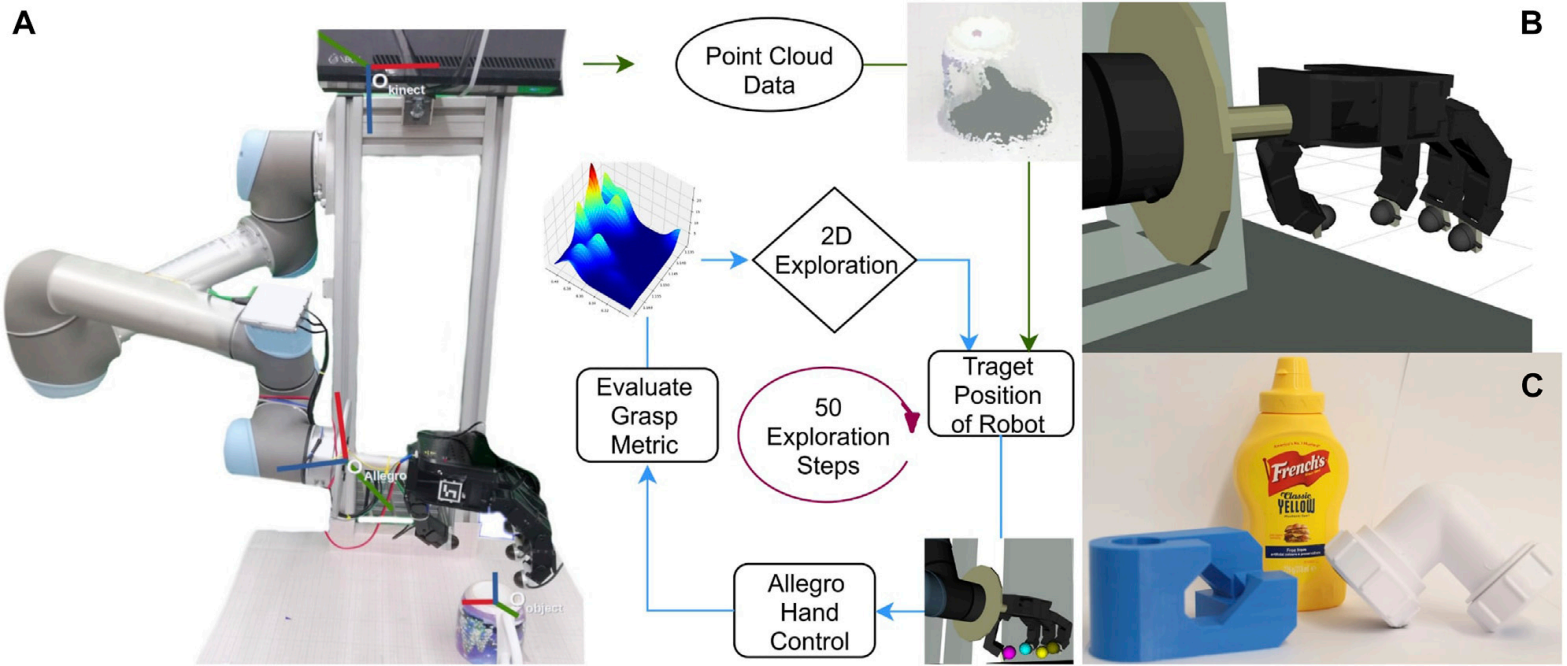
**(A)** Overview of the setup and the exploration pipeline. The setup includes a UR5 robot arm, an Allegro robot hand equipped with Optoforce 3D force sensors in the fingertips, a Kinect RGB-D sensor. **(B)** The Allegro hand visualised with the RVIZ software. **(C)** The three complex-shaped objects used in the experiment.

Robot perception for grasping typically includes vision, touch and proprioception; notably, all these modalities provide useful information about different aspects of the grasping problem. Vision is often the dominant modality in the phases that precede the lifting of the grasped object (Du et al., 2019), due to the ability to capture global information about the scene. However, vision is not equally effective at detecting local information about the interaction between the robot hand and the object, including the forces exerted by the hand, the hand configuration, and some physical attributes of the object, such as its stiffness or the friction coefficient of its surface: all these aspects are better perceived by touch. Therefore, the use of tactile sensing has become more and more popular (Luo et al., 2017), not just during the holding of the object (e.g. to react to slips) but also to discover how to grasp the object. In addition, the concept of active perception (Bajcsy et al., 2018), or interactive perception (Bohg et al., 2017), is particularly relevant in this case, because to collect useful tactile information the robot should perform relevant actions (Seminara et al., 2019), e.g. a controlled manual exploration of the object surface. However, one big challenge of relying on active real-time perception is the underlying uncertainty of robotic sensing and action generation (Wang et al., 2020). To cope with this uncertainty, we propose to enrich the visual information with a haptic exploration procedure driven by a probabilistic model, i.e. Bayesian Optimisation. The robot first detects the object location using point cloud data extracted from an RGB-D sensor. Then, an exploration procedure starts in which the robot hand evaluates different grasp configurations selected by Bayesian Optimization, based on a grasp metric computed from tactile sensing (Figure 1A). Finally, after the best grasp configuration is found, the object is picked up. We assume that the object is completely unknown to the system: we do not rely on any model or previous learning, but only on a real-time exploration that is relevant only for the current object and for the current execution of the grasp (i.e. not for any other object or any future execution of the grasp).

We extend the simulation results previously obtained in (Nogueira et al., 2016; Castanheira et al., 2018) by testing the system with a real robot hand, and by performing experiments on three objects with complex shapes. Notably, many additional uncertainties are present in a real-world environment (e.g. insensitivity of sensors, disturbance in the position of the object while exploring) that are not present in a controlled simulated environment. In particular, we show that an unscented version of Bayesian Optimization proves to be even more effective than the classic Bayesian Optimisation to discover robust grasps under uncertainty, with a limited number of exploration steps.

The contributions of the paper are threefold:

1) an approach to predict a safe grasp for an unknown object from a combination of visual and tactile perception.
2) a probabilistic exploration model that considers uncertainties of the real world in order to predict a safe grasp.
3) a series of experiments that demonstrate how the proposed system can find robot grasps that maximise the probability of the object being stable after it has been picked and lifted.

The paper is organised as follows: in Section 2, we describe the state of art for visuo-tactile data fusion and grasping of unknown objects. Section 3 provides an overview of our methodology. In Section 4, we describe the configuration and the experimental protocol. Discussion on the results is presented in Section 5. Finally, in Section 6, we conclude by summarising the performance of our approach and presenting possible directions for improvements and future research.

## 2 Related Work

Grasping objects of unknown shape is an essential skill for automation in manufacturing industries. Many existing grasping techniques require a 2D or 3D geometrical model, limiting its application in different working environments (Ciocarli and Allen, 2009). 3D reconstruction framework for detection of fruit in real environments is presented by Lin et al. (2020). Vision technology has advanced to detect objects in a natural environment over the years, even in the presence of shadows (Chen et al., 2020). Kolycheva née Nikandrova and Kyrki (2015) introduces a system using RGB-D vision to estimate the shape and pose of the object. The models for grasp stability are learnt over a set of known objects using Gaussian process regression. While 3D vision technology has various applications in the engineering field, acquiring 3D images is an expensive process and mostly simulation-based (Shao et al., 2019).

Merzić et al. (2018) makes use of deep reinforcement learning technique to grasp partially visible/occluded objects. It does not rely on the dataset of the object models but instead uses tactile sensors to achieve grasp stability on unknown objects in a simulation. Zhao et al. (2020) implements probabilistic modelling with a neural network to select a group of grasp points for an unknown object. There is also a work on learning object grasping based on visual cues, and the selection of features are often based on human intuitions (Saxena et al., 2008). However, vision-based accuracy is limited due to its standardization and occlusions. Some details can be overlooked even for known objects, which may cause failure in grasping objects (Kiatos et al., 2020). Our work is different from deep learning or reinforcement learning as there is no training data or an existing dataset to predict stable regions. The method explores an unknown object in real-time and finds a solution that maximizes a given grasp metric.

Tactile sensing is capable of compensating for some of the problems of the vision-only approach. Indeed, being able to perceive touch allows the robot to understand when contact with the object has been made and have a better perception of the occluded areas of the object by making contact with those surfaces. Techniques are proposed to control slippage and grasp stabilization of the objects using tactile sensors only (James and Lepora, 2020; Shaw-Cortez et al., 2020). It is independent of the data of object mass, object centre of mass and forces acting on the object to prevent the object from slipping. Rubert et al. (2019) present seven different kinds of grasp quality metrics to predict how well it performs on the robotic platform and in simulations. Different classifiers are trained on the extensive database, and results are evaluated for each grasp. The human labelled database is used in this work, which requires more accuracy in collecting data using different protocols. To accomplish the autonomous grasping of an unknown object, we aim to predict the grasping stability of the object before lifting the object from the surface. In this paper, we used tactile feedback to predict the stability of the robotic grasp. We present real-time grasp safety prediction by haptic probabilistic modelling exploration with a dexterous robotic hand.

The conventional methods address the stability of the objects during in-hand manipulation. Our method predicts the stability of the grasp before lifting the object off from the surface. Li et al. (2014) maintains the stability of the object grasped in the air by changing the grasp configuration of the robotic hand. The state of the object is disturbed by adding extra weight on the object or manually pulling the object from the grasp. The work of Veiga et al. (2015) focuses on slip detection using tactile sensors during in-hand manipulation. The main difference of our methods to existing approaches in the literature is that we do not use any previous learning/training on any object: all the search is performed in real-time on completely unknown objects, i.e. no prior information and no prior data is used.

## 3 Methodology

A self-supervised model is used to compute the probability of grasp success using tactile and visual inputs. This allows evaluating the robustness of potential grasps.

### 3.1 Object Detection

**FIGURE 2:**
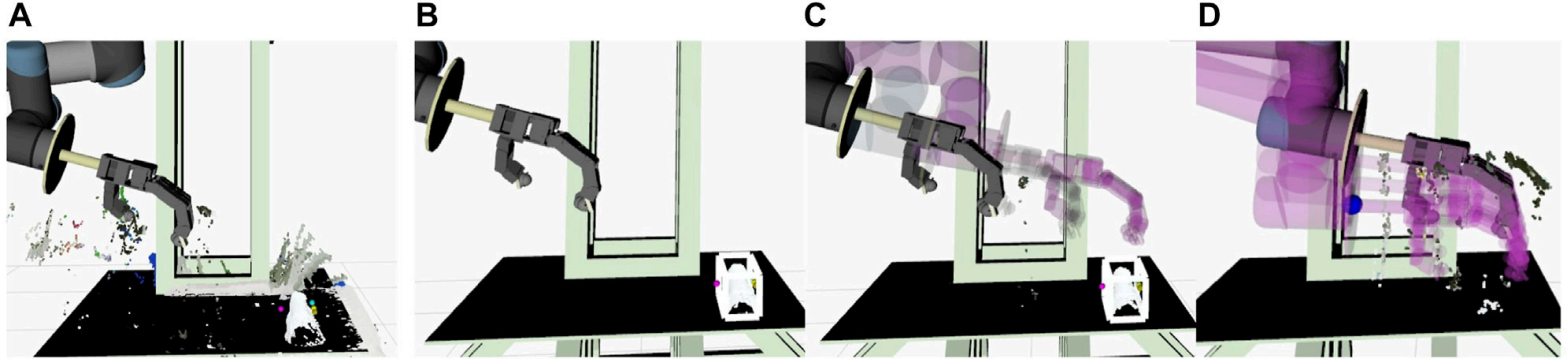
Methodology for the haptic exploration. **(A)** Point cloud data of the workspace. **(B)** The bounding box of the object extracted. **(C)** First step of path planning: towards a location at a fixed distance over the object bounding box. **(D)** Second step of path planning: lowering down and enclosing the fingers on the object to compute the grasp metric.

We use 3D point cloud data to calculate the midpoint of the object. We define a specific area in an environment as a workspace in which the robot operates safely. The robot perceives the object placed on the workspace while the remaining point cloud data is filtered out, as shown in Figure 2A.

We are using Random Sample Consensus (RANSAC), a non-deterministic iterative algorithm for detection of the object (Zuliani et al., 2005). It tries to fit the points from the point cloud into a mathematical model of a dominant plane. RANSAC then identifies the points which do not constitute the dominant plane model. These points that do not fit into the plane model (called outliers) are clustered together to form one object. A minimum threshold is set to avoid the detection of tiny objects and filtering extra noise. We demonstrate our approach only on singulated objects, i.e. not in clutter. The approach could be applied to clutters, but it would require more sophisticated visual perception components to segment each object and identify its boundaries partially.

Dimensions of the object are used to create a 3D bounding box around the object, as shown in Figure 2B. The midpoint of the object is computed as the difference between the maximum and minimum boundary points in an axis parallel to the plane. This point is then used to reference the robot to move close to the object and initiate tactile exploration. Path planning towards the object is executed in two steps to avoid collision with the environment. In the first step (Figure 2C), the arm moves to a safe distance above the object. The second step of path planning is then to move closer to the object (Figure 2D). Moveit! framework (Coleman et al., 2014) is utilised for implementation of motion planning. The process of instructing the robot to align itself closer to the object is described in Algorithm 1. The target pose is saved before the movement of the arm towards the object to avoid end-effector blocking the target during execution.

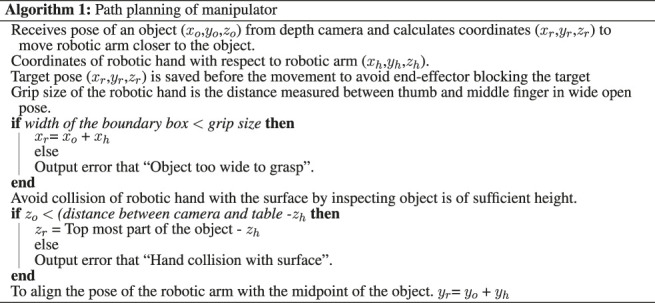

### 3.2 Force Metric Calculation

A constant envelop force is useful for the computation of force metric. An extensive review of the different criteria used for computing a grasp metric is described in Roa and Suárez, 2015. Following variables can be taken into account in the evaluation of a grasp metric:

• coordinates of the grasp points on the object.
• directions at which the force is applied at the grasp point.
• magnitude of the force experienced at the grasp point.
• pose of the robotic hand (in our case, Allegro hand).

Tactile exploration consists of closing the robotic hand at multiple points in an object and evaluating the grasp metric. In a closing state, fingers stop moving when the fingertips get in contact with the object. A force vector is created to grasp the object during metric calculation and picking the object. This force is calculated from coordinates of fingertip to virtual frame positioned in the middle of fingers and thumb. The concept of virtual springs is discussed in detail by Solak and Jamone (2019). Equation of grasp force is:

$$K_{i}\left(\left|\text{Δ}p_{i}\right|-L_{i}\right)\frac{p_{i}}{\left|\text{Δ}p_{i}\right|}$$ (1)

where $\text{Δ}p_{i}$ is vector between coordinates of fingertip and virtual frame. $K_{i}$ and $L_{i}$ are the stiffness and rest length of the spring, respectively.

The volume of the Force Wrench Space (FWS) by Miller and Allen (1999) is used as a force metric to measure the stability of the grasp during tactile exploration. FWS is defined as the set of all forces applied to the object with all grasp contacts. It is a three-dimensional vector consisting of force components from all the four tactile sensors positioned on the tip of the fingers of the robotic hand. This metric is also independent of the coordinates of reference system. Function $Q_{v}$ for this set of FWS (℘) can be described as:

$$Q_{v}=Volume\left(℘\right)$$ (2)

During the closing state, the robotic hand wounds its fingers around the object. The grasp metric is calculated when a connection is established between the hand and the object. The size and coordinates of the object are assumed fixed to limit the size of the exploration space.

### 3.3 Probabilistic Modelling

We use two probabilistic exploration methods: scented and unscented bayesian optimization, and compare their performance with uniform grid exploration. The uniform grid approach is where all search points in bounded space have an equal probability of being explored.

#### 3.3.1 Bayesian Optimisation

We consider the Bayesian Optimisation (BO) algorithm as one of the probabilistic models to accomplish the task of exploring global optima (Brochu et al., 2010). For n number of iterations, the input dataset of query point is *x* = {$x_{1:n}$} and the resulted outcome is *z* = {$z_{1:n}$}. In general, the algorithm depends on tuning parameters where input x *ϵ* X in some specified domain, where X ⊆ ${\mathbb{R}}^{D}$ , such that D ≥1. The main goal is to find the global optimisation method, which focuses on finding the minimum optimum value for the objective function f:X→ℝ, where X is a compact space. It works on selecting the best grasp points for every iteration geared towards the minimum $\left|z^{\text{*}}-z_{n}\right|$. Consider this process in two basic steps: First, for each grasp point input, a probabilistic model (in our case, the Gaussian process) is built. Second, using an acquisition function *α* to decide the model to select the next point for exploration. As the method depends on the trial-and-error approach, BO helps optimise the number of steps required for a safe grasp. Grasp metric score is computed as described in Section 3.2.

#### 3.3.2 Unscented Bayesian Optimisation

Unscented Bayesian Optimisation (UBO) is a method to propagate mean and covariance through nonlinear transformation. The basis of the algorithm is better manageability of an approximate probability distribution than approximate arbitrary nonlinear function (Nogueira et al., 2016). To calculate mean and covariance, a set of sigma points are chosen. These sigma points are deterministically chosen points that depict certain information about mean and covariance. The weighted combination of sigma points is then passed through linear function to compute the transformed distribution. The advantage of UBO over classical BO is it’s ability to consider uncertainty in the input space to find an optimal grasp. For dimension d, it requires 2d + 1 sigma points that show its computational cost is negligible compared to others such as Monte Carlo, which requires more samples or Gaussian function.

In UBO, the query is selected based on probability distribution. We choose the best query point considering it as deterministic, but also check its surrounding neighbours. Thus, while considering input noise, we will analyze the resulting posterior distribution through the acquisition function. Assuming that our prior distribution is Gaussian distribution where x∼N(barx,∑x), then the set of 2d + 1 sigma points of the unscented transform is computed as:

$$x^{0}=\bar{x},x_{\pm }^{i}=\bar{x}\pm {\left(\sqrt{\left(d∔\kappa \right)\sum x}\right)}_{i},\forall i=1...d$$ (3)

where d is dimensional input space, *κ* parameter tunes magnitude of sigma points and ${\left(\sqrt{\left(.\right)}\right)}_{i}$ is the _*i*_ *th* row or column of the corresponding matrix square root. UBO reduces the chance that the next query point is in an unsafe region where a small change in input results in a bad outcome.

## 4 Implementation

The grasp metric of a candidate grasp is evaluated on a real robotic platform. We start from elementary visual perception, which is used by the robot to come closer to the object and to be able to initiate the haptic exploration. Motion planning is initially visualised using the robot operating system (ROS) before execution in the real-world environment. Experiment to pick the object from the surface is designed to the evaluate performance of the exploration algorithm. Objects are manually put in the same approximate location to maintain consistency in the evaluation and show that the grasps found with UBO are more resilient to minor variations in object position.

In a real use-case, the robot hand would approach the object (starting from the visual estimation of the object pose). It would haptically explore the object (without lifting it, only by touching it in the different possible grasp postures/configurations) to maximise a given grasp metric (i.e. based on the measured contact forces), and then it would lift the object by using the best grasp that has been found with the haptic exploration. This is relevant for scenarios in which we want to optimise the safety of the grasp over speed, e.g. nuclear-decommissioning settings, or other scenarios in which we do want to minimise the possibility of the object falling from the grasp, at the cost of requiring more time to find the safest grasp.

### 4.1 Configuration

For the experimental setup, a camera is required to generate point cloud data of the objects. The generation of the point cloud can be achieved using a stereo camera or RGBD camera. The authors in Vezzani et al., 2017 have used a stereo camera to generate point cloud data, and authors of Rodriguez et al., 2012 are using Kinect. Both have presented that the generated point cloud is satisfactory so that any camera can be selected. We have used a kinect camera for the generation of point cloud data in our experiments.

To achieve our objective of successfully grasping an unknown object, we have set up a UR5 robot in the lab. Allegro hand is mounted at the end of the UR5 arm as an end effector. Kinect is fixed at the top of the base of the robot, facing perpendicular to the workspace. Optoforce OMD 20-SE-40N is a 3-axis force sensors that measure the forces experienced by the fingers of the Allegro hand (at a rate of 1 kHz). The workplace is 72 cm from the kinect frame. Any object within the workplace area (a rectangular area of 31 cm by 40 cm) is processed, and the extra points are filtered out. The orientation of the Allegro hand is fixed parallel to the axis of the workspace plane. The setup is shown in Figure 1A.

### 4.2 Protocol

To perform the experiments, we apply the following experimental protocol.

1) Object detection: to detect the unknown object in the environment, we use the RANSAC algorithm in point cloud library. This library allows the detection of the desired object and obtains its pose with respect to the camera.
2) Motion planning: once we have detected the pose of the object, the Moveit plans the collision-free movement of the robot to the top of the object.
3) Plan execution: after successful planning, the robot navigates itself to the target pose. This is also the starting pose for haptic exploration.
4) Haptic exploration: robot plans and navigates the robotic hand to search points queried by the exploration model. Search space is confined by limiting the orientation of the Allegro hand parallel to the surface.
5) Gradually gripping the object: when the robotic arm reaches the search point, it starts closing its fingers until contact is detected.
6) Applying grasping force: to ensure the gripper applies enough pressure over the object and not just touches it.
7) Calculation of grasp metric: evaluate grasp score of the candidate grasp.
8) Move the robotic arm to the next pose: open the grip of the robotic hand and move to the next pose directed by the probabilistic model. This process is repeated 50 iterations. Approximately two iterations are completed in a minute.
9) Stability testing: this experiment is performed after the completion of the exploration stage. The robot is manually navigated to the coordinates of the maximum grasp metric score to evaluate its stability.
10) Stability scoring: object is lifted 20 times from the surface and maintained in the air for 10 s.

## 5 Results

The proposed model is validated by exploring grasp points in the 3D space, but the contact points are searched on two dimensions. Experiments are conducted five times with probabilistic modelling exploration and then compared with the uniformly distributed exploration. BO and UBO models are used for probabilistic modelling exploration. At the end of each experiment, grasp point with the highest metric is used to lift the object from the surface. This process of lifting the object is repeated 20 times to find the stability score of the grasp point.

We used the objects from the dataset^1^ developed by EU RoMaNS to observe exploration performance. The objects in this dataset are commonly found in nuclear waste and are categorised in different categories such as bottles, cans, pipe joints. We conducted the experiments with three different kinds of complex objects: a c-shaped pipe joint, a mustard plastic bottle and a 3D printed blue object. The diameter of pipe joint is *6 cm* from one end and *5.5 cm* from another, the height of thread on the ring is *0.2 cm*. A complex-shaped 3D printed blue object *11cm × 5.5 cm* from dexnet dataset^2^ is also used to increase the persuasiveness of the data. Images of the objects can be seen in the Figure 1C. Objects were placed on the bubble wrap surface to increase the friction between the object and the plane. This was done because the fingertip force sensors are not very sensitive, and therefore the minimum contact force that can be measured (at first contact) may already produce a consistent displacement of the object (if the friction coefficient of the table surface was too low).

**FIGURE 3:**
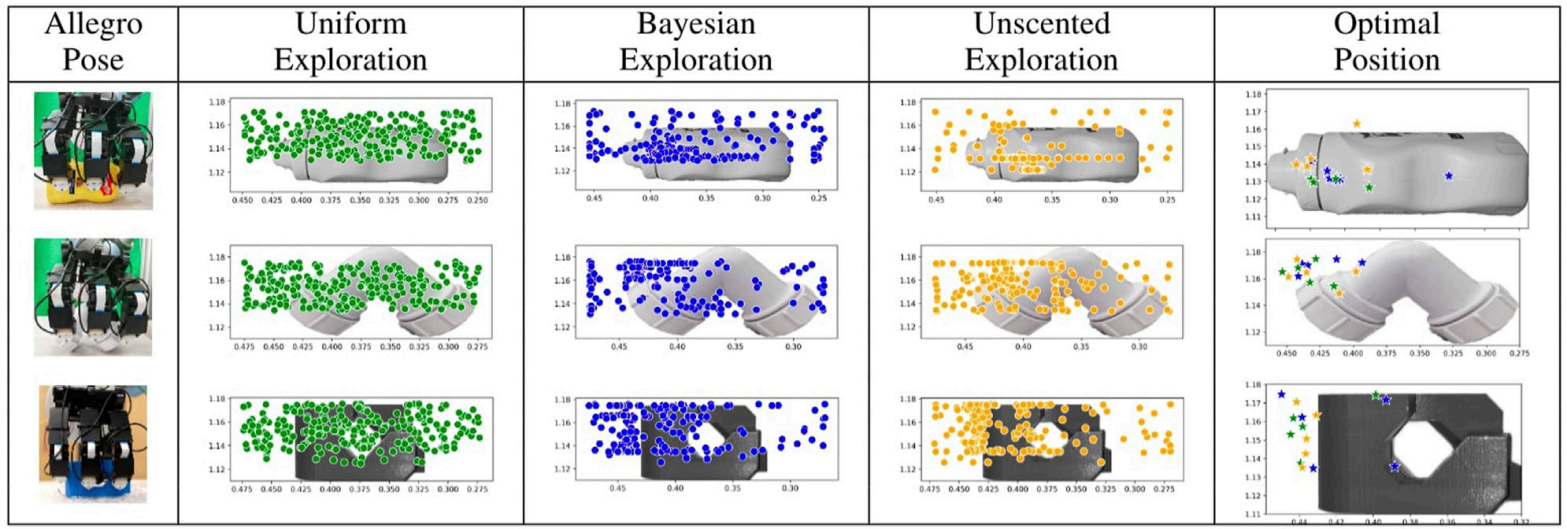
Scatter Plots of all points explored in uniform, BO and UBO explorations for **(A)** bottle, **(B)** pipe joint and **(C)** blue object. The pose of Allegro hand at the start of experiments is shown in the first column. The last column represents the optimum position in 2D from each experiment.

**Scatter plots:** The Figure 3 represents the points observed by each exploration method in all the experiments. The point represents the location of the middle finger of the robotic arm. A total of 250 search points (5 experiments with 50 iterations each) are plotted for each exploration method. It can be observed that more observations are recorded at the boundaries of the object for probabilistic methods. This is due to the concavity of the tactile sensors and their contact with the edges in the objects. It is expected for probabilistic models to explore the complex part of the object. It can also be seen that BO and UBO exploration converges to a more substantial part of the object. The figure also represents the optimal position with the highest metric score for all experiments for each exploration model. There are a total of 15 points represented, five for each approach. Again, the points are the location of the middle finger of the robotic arm.

**Optimal position:** The position with optimal grasp score is the distance from the world frame along the horizontal plane of the object. The middle point of the bounding box of the bottle is approximately 35 cm from the world frame and 37.5 cm in the pipe joint and blue object. The frames are shown in Figure 1A. Table 1 tabulates the optimal position of the object as observed in each experiment. It also shows the value of grasp metric value in the optimal position. The points are skewed towards one side of the object because of the constraint in the encoders of the thumb, which restricts the movement of the thumb to align with the middle finger (Figure 1B). The results indicate that probabilistic models have an optimum position similar to uniform distributed exploration with minor standard deviation in position and metric score.

**TABLE 1:** Coordinates of maximum metric observed of explorations for different objects from all experiments in the world frame. Standard deviation of the mean position in *x* and *y* axes, metric is also listed.

	Bottle			Pipe joint			Blue object		
	UNI	BO	UBO	UNI	BO	UBO	UNI	BO	UBO
*μ* X-cord (cm)	39.01	37.34	39.07	43.44	42.41	42.71	43.29	42.05	43.64
*σ* X-cord (cm)	1.64	2.65	0.67	1.3	1.84	1.94	1.72	2.49	0.34
*μ* Y-cord (cm)	114.43	113.27	113.12	116.51	117.03	116.34	115.68	115.58	115.25
*σ* Y-cord (cm)	0.97	0.19	0.08	0.68	0.43	0.83	1.21	1.73	1.3
*μ* Grasp metric	27.49	30.22	29.1	35.08	39.64	37.13	36.37	37.49	35.63
*σ* Grasp metric	1.51	2.6	0.48	6.7	3.78	2.82	3.3	6.63	1.77

**Convergence:** The convergence of each exploration to its maximum grasp metric value reflects confidence in successfully lifting the object from the surface. The left column of Figure 5 presents the performance of BO, UBO and uniform explorations in converging to the final optimum position (*x*-axis) at each observation. Uniform, BO and UBO are represented by green, blue and orange lines, respectively. A total of five experiments are conducted with 50 observations for three different complex-shaped objects. Plots present convergence in the *x*-axis only because of the confined range of exploration in the *y*-axis (<±4cm). It can be seen that the probabilistic models have a higher probability of convergence than the uniform-grid search model. There are some instances when convergence is not observed; this is understandable as the number of iterations is very low.

**FIGURE 4:**
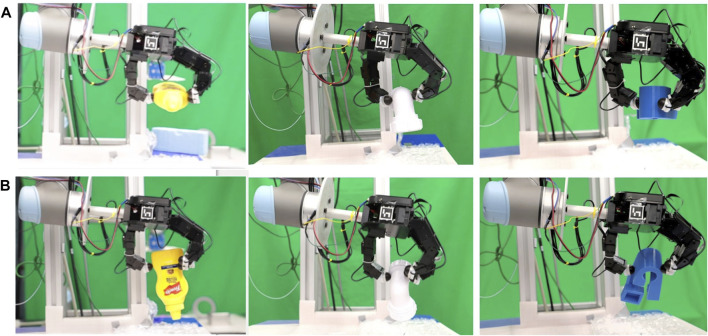
Two states of stability observed while maintaining the object in the air: **(A)** stable grasp **(B)** partial stable grasp.

**FIGURE 5:**
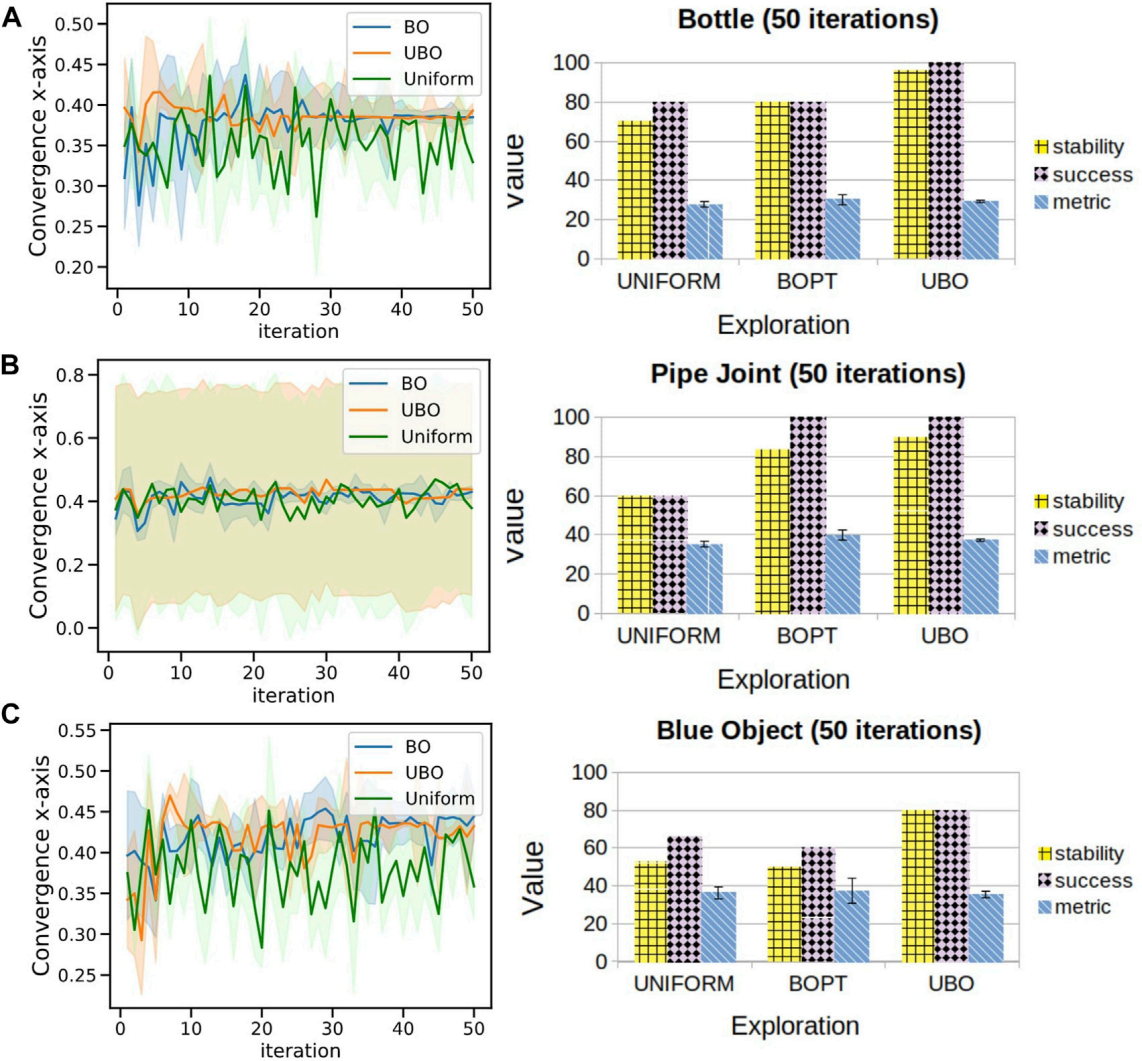
Left column displays performance of uniform, BO and UBO explorations in converging to final optimum position (*x*-axis) in all experiments for **(A)** bottle, **(B)** pipe joint and **(C)** blue object. Right column shows stability scores and mean of the highest grasp metric for different exploration methods.

**Stability score:** There are three possible states of stability when the object is lifted in the air: stable, partial stable and failure. A stable state is when three or four fingers of the robotic hand contact the object, and the object stays in the air for 10 s. Partial stability is when only the thumb and first finger hold the object in the air for 10 s. These two states are shown in Figure 4. Failure state is when the robotic arm fails to lift the object off the surface. In none of our experiments, an object dropped from the air. Table 2 tabulates the performance of each exploration in lifting the object. The results of the experiment to evaluate stability is shown on the right column of Figure 5. Frequency distribution of the five experiments for objects used is tabulated in Tables 3–5. The success rate for each exploration is the percentage of the robot lifting the object from the surface (both stable and partial state) and holds it in the air without a drop-off. In the calculation of stability score, the stable state is given double weight than the partial state. Failure state is excluded from the calculation. The formula is mentioned below:

$$\mathrm{Stability}\;score=\frac{Stable\;*10\;+\;Partial\;*5}{Total\;possible\;score}\times 100$$ (4)

**TABLE 2:** Grasping state observed for all five experiments in lifting the object from surface. Total of five experiments with 20 iterations each.

	Bottle			Pipe joint			Blue object		
	UNI	BO	UBO	UNI	BO	UBO	UNI	BO	UBO
Total stable	60	80	92	60	67	80	40	40	80
Total partial	20	0	8	0	33	20	26	20	0
Total failure	20	20	0	40	0	0	34	40	20
Success rate (%)	80	80	100	60	100	100	66	60	80
Stability score	70	80	96	60	83.5	90	53	50	80

**TABLE 3:** Frequency distribution of experiments on bottle to evaluate grasp stability.

	Uniform			BO			UBO		
	Stable	Partial	Fail	Stable	Partial	Fail	Stable	Partial	Fail
Exp 1	0	20	0	20	0	0	20	0	0
Exp 2	20	0	0	20	0	0	20	0	0
Exp 3	20	0	0	0	0	20	20	0	0
Exp 4	0	0	20	20	0	0	12	8	0
Exp 5	20	0	0	20	0	0	20	0	0

**TABLE 4:** Frequency distribution of experiments on pipe joint to evaluate grasp stability.

	Uniform			BO			UBO		
	Stable	Partial	Fail	Stable	Partial	Fail	Stable	Partial	Fail
Exp 1	20	0	0	7	13	0	20	0	0
Exp 2	0	0	20	20	0	0	20	0	0
Exp 3	0	0	20	20	0	0	20	0	0
Exp 4	20	0	0	0	20	0	20	0	0
Exp 5	20	0	0	20	0	0	0	20	0

**TABLE 5:** Frequency distribution of experiments on complex-shaped blue object to evaluate grasp stability.

	Uniform			BO			UBO		
	Stable	Partial	Fail	Stable	Partial	Fail	Stable	Partial	Fail
Exp 1	0	9	11	0	20	0	20	0	0
Exp 2	20	0	0	0	0	20	20	0	0
Exp 3	20	0	0	20	0	0	20	0	0
Exp 4	0	0	20	20	0	0	20	0	0
Exp 5	0	17	3	0	0	20	0	0	20

The results show that probabilistic models can converge to the optimum position with a higher grasp metric score in fewer iterations than uniformly distributed exploration.

The experimental results collected demonstrate:

• the ability of probabilistic methods to provide confidence in predicting a safe grasp in a minimal number of iterations.
• BO and UBO have the advantage of converging sooner than the uniform exploration, even with fewer observations.
• the potential of UBO to find safer grasps: this is evident in the case of the bottle, as the optimum points lie far from the edges.
• the success rate of UBO is the highest in lifting the object from the surface and maintaining it in the air for 10 s (i.e. stable grasp).

## 6 Conclusion

We presented a pipeline for object detection (using depth-sensing) and exploration (using tactile sensing) with a dexterous robotic hand, aimed at finding grasps that maximise the probability of the object being held robustly in hand after picking and lifting. Our approach is not based on any previous learning or prior information about the object: the system knows nothing about the object before the exploration starts.

The intelligence of the system lies in the real-time decisions about where to explore the object at each exploration step, so that the number of exploratory steps is minimised and the amount of information gathered is maximised. These decisions are based on a probabilistic model (BO). In particular, we show experimentally that an unscented version of the model (UBO) can find the more robust grasps, even in the presence of the natural uncertainty of robotic perception and action execution: we show this by repeating the grasps multiple times, showing that such grasps are robust to the minor inaccuracies/differences between each replication of the grasp. Given the nature of this approach, the most relevant applications are in scenarios in which the cost of dropping the object after grasp is very high, and it is therefore justified to invest some additional time in exploring the object haptically before picking it. For example, handling hazardous materials in a nuclear environment, collecting samples in space or deep sea missions, pick and place of fragile objects in logistics.

In our experiments, we assume to have no prior knowledge about the object; however, such information (if available) could be included in the probabilistic exploration models as a prior, also depending on the specific application. We use depth sensing to limit the search space by identifying a bounding box around the object: a more sophisticated visual perception component could permit defining an even more compact search space, e.g. consisting in a small set of tentatively good grasps.

Another possible improvement of our system is to use better tactile sensors on the robot fingertips that are more sensitive (Jamone et al., 2015; Paulino et al., 2017) and that can provide 3D force measurements on several contact points (Tomo et al., 2018). With such a sensor: we could detect the initial contact with the object earlier (i.e. based on a lower force threshold), therefore minimising undesired motion of the object during exploration; we could obtain a better estimation of the contact forces, that would lead to a more reliable assessment of the force closure metric; we could estimate other object properties (e.g. friction coefficient) that can also be included in the grasp metric, leading to better predictions of the grasp stability.

## Data Availability

Publicly available datasets were analyzed in this study. This data can be found here: https://github.com/ARQ-CRISP

## Footnotes

1
https://sites.google.com/site/romansbirmingham

2
https://berkeley.app.box.com/s/6mnb2bzi5zfa7qpwyn7uq5atb7vbztng
